# Case Report: Recurrent phyllodes tumor of the breast with progressive malignant transformation

**DOI:** 10.3389/fonc.2025.1652370

**Published:** 2025-12-16

**Authors:** Chuanyan Wang, Shangui Wang, Jiao Wang, Ya Dong, Yuhua Chi, Yanbing Wang

**Affiliations:** 1 Department of Oncology, Rizhao People’s Hospital, Rizhao, China; 2 Department of Pathology, Rizhao People’s Hospital, Rizhao, China; 3 Department of Nuclear Medicine, Rizhao People’s Hospital, Rizhao, China

**Keywords:** phyllodes tumor of the breast, recurrence, malignant transformation, treatment, case report

## Abstract

Phyllodes Tumor of the Breast (PTB) is a rare fibroepithelial tumor often misdiagnosed as fibroadenoma, leading to delayed or incorrect treatment. Although most PTBs are benign, some cases exhibit recurrent growth and progression to malignant forms. This report presents a 52-year-old female patient with PTB, who experienced multiple recurrences since her initial surgery in 2012, with the pathological grade gradually progressing from benign to borderline and ultimately malignant, characterized by heterologous osteosarcomatous differentiation. The tumor showed high heterogeneity and rapid progression. The final diagnosis was malignant phyllodes tumor of the breast with focal osteosarcomatous stromal components. Immunohistochemistry revealed a significant increase in the Ki-67 index from 8% to 70%, MDM2 positivity, and abnormal p53 expression, suggesting molecular transformation mechanisms. The patient underwent a modified radical mastectomy and three cycles of postoperative systemic chemotherapy with a combination of ifosfamide and cisplatin, which was well-tolerated. Thirty-six months after surgery, there was no recurrence or metastasis, and the disease remained stable. This case highlights the progression of PTB from benign to malignant and suggests that elevated Ki-67 and abnormalities in MDM2/p53 could serve as potential molecular markers. For patients with multiple recurrences or heterogeneous PTB components, individualized treatment strategies are recommended, with reference to soft tissue sarcoma protocols to optimize prognosis.

## Introduction

Phyllodes Tumor of the Breast (PTB) is a rare fibroepithelial breast tumor, representing 0.3%-1% of all breast tumors (1). Characterized by its leaf-like appearance on cross-section, PT is primarily composed of fibroblasts and mammary epithelial cells (2). It typically grows rapidly, presenting as one or more masses with some degree of local invasiveness. The World Health Organization classifies PT into benign, borderline, and malignant categories based on its malignancy (3). Malignant PT has a significantly higher risk of local recurrence (23%-30%) and metastasis (10%-20%) (4). PT most commonly affects women around 40 years of age (5). Although most cases are benign, the potential for recurrence and malignant transformation complicates clinical management (6, 7). Diagnosis involves imaging (ultrasound, mammography) and pathological evaluation but is often confused with fibroadenomas, leading to a high misdiagnosis rate (8). Surgical excision remains the primary treatment, though the optimal surgical margin for borderline and malignant PT, as well as the role of adjuvant therapies such as radiotherapy, remains debated (9). The molecular mechanisms underlying PT are not well understood, and reliable biomarkers to predict recurrence and malignant transformation are lacking. This article reports a case of a recurrent PT with progressively worsening pathological grades, aiming to explore its clinicopathological features and treatment strategies, thereby providing a foundation for personalized management of rare tumors.

## Case presentation

### Patient information

**Figure 1:**
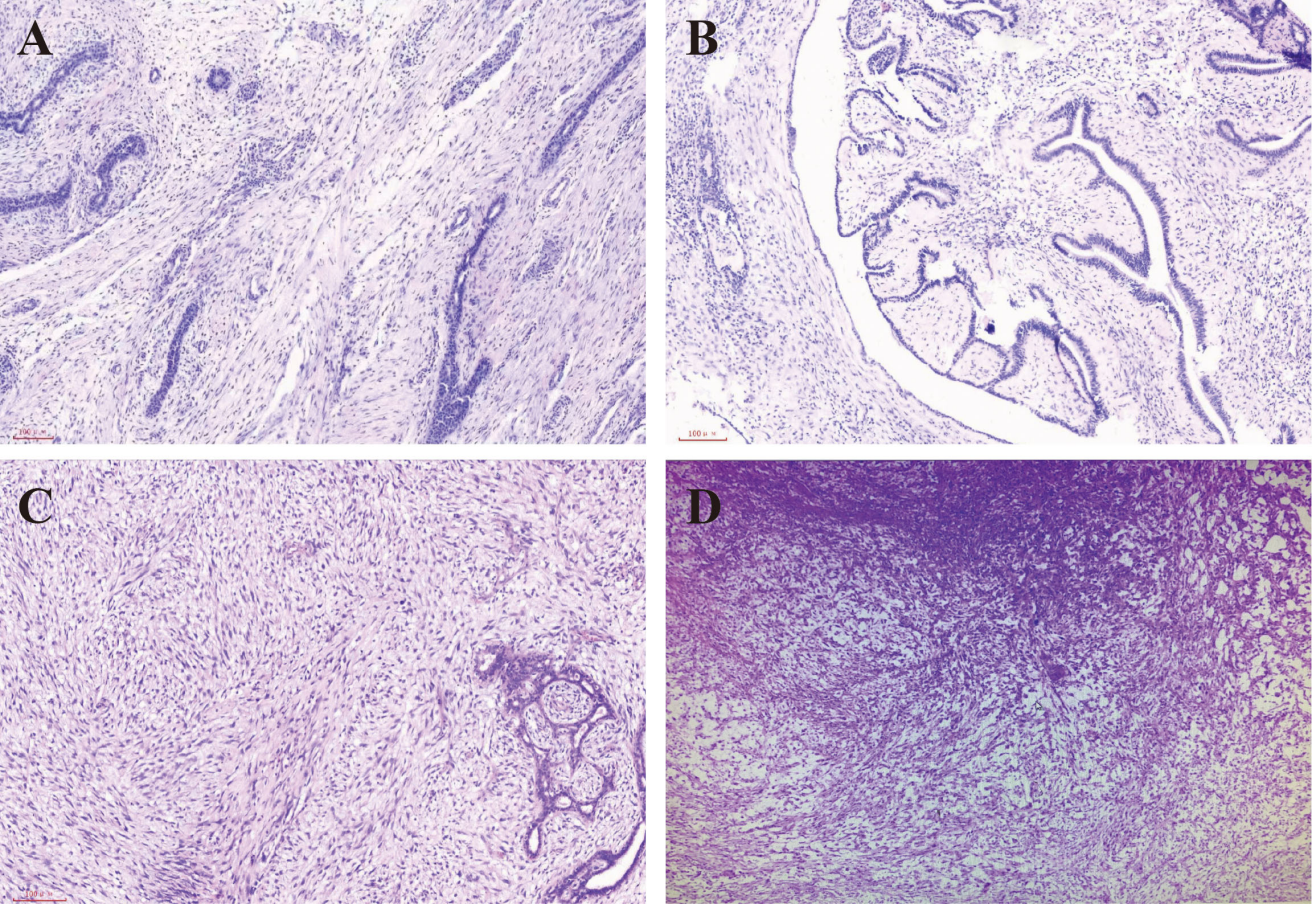
**(A)** HE staining of the specimen from January 22, 2013, showing a benign phyllodes tumor with well-defined borders, lobulated shape, and no malignancy at ×100 magnification. **(B)** HE staining of the specimen from May 25, 2018, showing a borderline phyllodes tumor with increased cellularity and mitotic figures (>10/10 HPF), indicating higher proliferative activity at ×40 magnification. **(C)** HE staining of the specimen from November 2, 2021, showing a borderline phyllodes tumor with areas of necrosis, inflammatory infiltration, and small vessel proliferation at ×100 magnification. **(D)** HE staining of the specimen from February 8, 2022, showing malignant phyllodes tumor with a high degree of stromal sarcomatous transformation and loss of epithelial characteristics at ×40 magnification.

A 52-year-old female presented in December 2012 with a walnut-sized mass in her left breast. The mass was firm, poorly mobile, and asymptomatic, with no nipple discharge or bleeding. Over time, the mass gradually increased in size. The patient has no family history of breast cancer, ovarian cancer, or other related diseases. Additionally, the patient has a history of hypertension. By January 12, 2013, a breast ultrasound revealed a 5.2×2.4 cm mass in the outer quadrant of the left breast, characterized by heterogeneous internal echoes, well-defined borders, and an irregular shape. A mammogram on January 17, 2013, confirmed the presence of a mass in the upper outer quadrant of the left breast, classified as BI-RADS 4B. On January 22, 2013, the patient underwent breast mass excision. Intraoperatively, the mass, located in the upper outer quadrant of the left breast, measured approximately 5×4×4 cm, was lobulated, firm, with clear boundaries and an intact capsule. Postoperative pathology (**Figure 1A**) confirmed the diagnosis of a benign phyllodes tumor, measuring 7×4.5×2.5 cm. The patient made a full recovery following the surgery.

### First recurrence

In May 2017, the patient noticed a mass in her left breast, approximately the size of a quail egg, but delayed seeking medical attention. She visited our hospital on May 21, 2018. Breast ultrasound revealed bilateral breast hyperplasia, with a solid mass in the upper outer quadrant of the left breast and a mixed cystic-solid nodule in the inner part of the left breast. Mammography showed a mass in the upper outer quadrant of the left breast, measuring approximately 6.6×6.6 cm, with well-defined margins, a lobulated shape, and a 1.9×1.9 cm nodule at the posterior outer region, classified as BI-RADS 4B. On May 25, 2018, the patient underwent partial mastectomy of the left breast. Intraoperatively, the mass measured approximately 6.0×6.0×6.0 cm, was firm, solid, with well-defined boundaries, and an intact capsule. Postoperative pathology (**Figure 1B**) confirmed the diagnosis of a borderline phyllodes tumor, with tumor sizes of 2.0×1.5×1.5 cm and 7.0×6.5×5 cm. The pathological report indicated that no tumor was present at the left breast surgical margins, and the margin status was negative. Immunohistochemistry showed Calponin(+), SMA(+), Bcl-2(-), CD34(-), ALK(-), with a Ki-67 index of 8%. The patient had a smooth recovery post-surgery.

### Second recurrence

In October 2020, the patient detected a left breast mass approximately the size of an egg but did not seek immediate medical attention. On October 31, 2021, she presented to our hospital. Breast ultrasound revealed a large mixed cystic-solid mass in the left breast measuring approximately 15 × 20 × 8.2 cm, with irregular shape, heterogeneous margins, fine internal echoes, and classified as BI-RADS 4B. Preoperative breast MRI indicated mild background degeneration in both breasts, with an irregular lesion in the left breast showing uneven signal intensity, slightly prolonged T1 and T2 signals, mild DWI elevation, and irregular ring enhancement post-contrast. The BI-RADS score was 4B-4C. The right breast showed no abnormal findings, with a BI-RADS score of 1.On November 2, 2021, a partial mastectomy of the left breast was performed. Intraoperatively, the tumor measured 16 × 15 × 10 cm, was solid and firm with a well-defined capsule, and was completely excised along with adjacent glandular tissue. Postoperative pathology (**Figure 1C**) confirmed a borderline phyllodes tumor (15.5 × 12 × 8 cm) with areas of fibrofatty tissue necrosis, acute and chronic inflammatory infiltration, and small vessel proliferation. The patient had a moderate postoperative recovery.

### Third recurrence and malignant transformation

In January 2022, the patient noted a recurrent mass in the left breast. Ultrasound on January 24 revealed multiple nodules, with the largest measuring approximately 4.27 × 2.38 cm at the 3 o’clock position beneath the nipple. The lesion appeared oval, with smooth margins and hypoechoic internal features, and was classified as BI-RADS 4A, suggestive of a phyllodes tumor. The patient was referred to Qilu Hospital of Shandong University for further evaluation. Pathology review of previous specimens confirmed a benign phyllodes tumor in 2013, a malignant phyllodes tumor in 2018 (with >10 mitoses/10 HPF and marked stromal overgrowth), and a malignant phyllodes tumor with heterologous osteosarcomatous differentiation in 2021. On February 8, 2022, the patient underwent a modified radical mastectomy under general anesthesia. Intraoperatively, the tumor was localized to the lateral region of the left breast. Postoperative pathology (**Figure 1D**) confirmed a malignant phyllodes tumor consistent with pleomorphic undifferentiated sarcoma, lacking epithelial components. Multifocal tumors were identified, with excised nodules measuring 2.2 × 1.5 cm, 4.7 × 3.5 cm, 4.1 × 3 cm, 3.2 × 2.5 cm, and 2.5 × 1.5 cm. No tumor involvement was observed in the nipple, skin, or surgical margins, and no vascular tumor emboli were detected. The remaining breast tissue showed fat necrosis, fibrosis, and foreign body granulomas. All 20 axillary lymph nodes were negative for metastasis (0/20). Immunohistochemistry revealed the following: Vimentin (+), CD68 (+), TFE-3 (+), MDM2 (+), CK (focal +), SATB2 (weak +), CDK4 (weak +), S-100 (partial +), SMA (partial +), P16 (partial +), P53 (+++, 10%), INI-1 (+), CD34 and CD31 (vascular endothelial +), Desmin (−), MUC4 (−), HMB45 (−), ALK (−), NUT (−), CD30 (−), ER (−), PR (−), HER2 (0). The Ki-67 proliferation index was 70%.

A summary of the patient’s clinical course, tumor features, treatment strategies, and pathological findings is provided in **Table 1**.

**Table 1:** Clinical timeline and pathological evolution of a recurrent phyllodes tumor case.

Time	Event	Tumor characteristics	Treatment	Pathological results
2012.12	Initial Lump	Walnut-sized, firm, poor mobility	–	–
2013.01	First Surgery	7×4.5×2.5cm, lobulated, hard texture	Tumor excision surgery	Benign phyllodes tumor
2018.05	First Recurrence	7.0×6.5×5cm, 2.0×1.5×1.5cm, lobulated, firm, clear boundary	Partial mastectomy	Borderline phyllodes tumor (Ki-67 8%)
2021.11	Second Recurrence	15.5×12×8cm, cystic-solid mixture	Partial mastectomy	Borderline phyllodes tumor (with necrosis, inflammatory cell infiltration)
2022.02	Third Recurrence (Malignant Transformation)	Multicentric, largest 4.27×2.38cm, firm, clear boundary	Modified radical surgery + Chemotherapy	Malignant phyllodes tumor (Ki-67 70%)

### Postoperative treatment and follow-up

Postoperatively, the patient underwent three cycles of combination chemotherapy between April 13 and May 29, 2022, comprising ifosfamide (2 g, days 1–5) and cisplatin (40 mg, days 1–3). Although postoperative radiotherapy was recommended to reduce the risk of local recurrence, the patient declined the treatment due to personal reasons. She was subsequently monitored through intermittent follow-up. At the most recent follow-up on May 6, 2025, breast ultrasound revealed no significant abnormalities in the left chest wall, no axillary lymphadenopathy, and no space-occupying lesions in the right breast. The disease was considered stable.

## Discussion

PTB is a rare fibroepithelial neoplasm, comprising less than 1% of all breast tumors (1). First described by Muller in 1838, its potential for malignant transformation was recognized in 1931. The World Health Organization (WHO) classifies PTB into three categories: benign (60%), borderline (20%), and malignant (20%) (3). While PTBs typically present as single, painless, well-defined masses, rapid growth, a size exceeding 3 cm, and atypical clinical features should raise suspicion for a phyllodes tumor.

The uniqueness of this case lies in its comprehensive documentation of the entire process of primary PTB evolution from benign to malignant, highlighting three key pathological turning points: (1) In 2018, the recurrence showed mitotic figures > 10/10 HPF, indicating a significant increase in cell proliferation activity; (2) In 2021, the emergence of heterologous osteosarcomatous components suggested differentiation shifts in the stromal components; (3) In 2022, the tissue completely lost its epithelial characteristics, with only small foci of CK positivity, indicating near-total disappearance of epithelial components. This dynamic progression challenges the traditional view that “the pathological type of recurrent PTB remains constant” and supports the hypothesis that some PTBs may be driven by dedifferentiation mechanisms due to cumulative genetic damage. Previous studies have linked the histological grade of PTB to molecular abnormalities, such as TERT promoter mutations, MED12 mutations, p53 expression abnormalities, and Wnt/β-catenin pathway activation, which may accumulate over time and promote tumor progression (10, 11). Additionally, the high heterogeneity of the tumor stroma may facilitate multidirectional evolution. Notably, in this case, the Ki-67 index continuously increased with the pathological upgrading (from 8% to 70%), suggesting a significant enhancement in tumor biological behavior. This observation aligns with the findings of Ashley Cimino-Mathews et al., who proposed that Ki-67 expression significantly increases in malignant PTB and correlates with tumor histological grade, making it a valuable adjunctive grading indicator (12).

The treatment of PTB is primarily surgical. For benign PTBs, breast-conserving surgery is typically performed, followed by regular follow-up. In the case of borderline and malignant PTBs, local wide excision is recommended. Surgery remains the first-line treatment for benign PTBs without distant metastasis. The role of adjuvant radiotherapy for borderline and malignant PTBs post-surgery remains a topic of debate. Although phyllodes tumors of the breast (PTB) predominantly metastasize via hematogenous routes, lymph node metastasis remains a critical consideration, particularly for large or highly invasive tumors. In this case, the patient’s PTB recurred multiple times and progressively underwent malignant transformation. Pathological examination in 2022 confirmed high tumor invasiveness. Consequently, axillary lymph node dissection was performed to rule out lymph node metastasis and to inform postoperative treatment decisions, including the potential need for chemotherapy or radiotherapy. A 2009 study by Barth et al. (13) found that patients with borderline and malignant PTBs, who had negative surgical margins and received postoperative radiotherapy, experienced no local recurrence after an average follow-up of 60 months (range: 12–129 months). Similarly, Boutrus et al. (14) demonstrated that adjuvant radiotherapy significantly improved the 5-year disease-free survival rate. In real-world studies, the proportion of borderline and malignant PTB patients receiving adjuvant radiotherapy has gradually increased, but there is no conclusive evidence that it improves overall survival. There is limited research on the need for adjuvant chemotherapy in malignant PTB patients. A multicenter retrospective study by Neron et al. (15) found no significant benefit from postoperative adjuvant chemotherapy in malignant PTB cases, and no reports have been published on adjuvant targeted therapy or endocrine therapy. Current expert consensus does not recommend adjuvant chemotherapy, targeted therapy, or endocrine therapy for primary malignant PTB. However, for patients with primary malignant phyllodes tumors of the breast at high risk for postoperative recurrence or distant metastasis, clinical management generally follows the treatment protocols for soft tissue sarcomas. Chemotherapy typically includes anthracycline-based regimens, such as doxorubicin combined with ifosfamide, or non-anthracycline alternatives like ifosfamide with cisplatin (16–18).

In this case, the patient underwent multiple surgeries and ultimately received modified radical surgery followed by chemotherapy with ifosfamide and cisplatin. Thirty-six months post-surgery, no disease progression has been observed. Although current guidelines do not recommend postoperative adjuvant chemotherapy for primary malignant PTB, the favorable outcome in this case suggests that patients with recurrent malignant PTB may benefit from more aggressive treatment strategies. This case report aims to provide additional clinical evidence on the diagnosis, treatment, and prognosis of phyllodes tumors. A primary area of controversy remains the use of adjuvant therapy for malignant PTB. Although studies by Barth (13) and Boutrus (14) have demonstrated that radiotherapy can reduce local recurrence rates, this case suggests that for patients with multiple recurrences and sarcomatoid transformation, systemic therapy may play a more pivotal role. The rationale for using ifosfamide and cisplatin chemotherapy includes: (1) the heterologous components of the tumor may be responsive to sarcoma chemotherapy regimens (19, 20), and (2) animal model studies have indicated that cisplatin can inhibit the growth of tumors with MDM2 overexpression (21). This interdisciplinary treatment approach warrants further investigation in prospective clinical trials.

This case also underscores the crucial role of early involvement by expert pathologists in the management of rare tumors such as phyllodes tumors (PTB). The challenges associated with the initial diagnosis highlight the necessity of professional pathological expertise for accurate diagnosis, grading, and malignancy assessment. Consequently, referring such cases to specialized centers for multidisciplinary team discussions is essential for formulating an optimal treatment plan and enhancing patient prognosis.

## Conclusions

This case provides the first comprehensive documentation of the pathological transformation of PTB from benign to malignant, including the emergence of heterologous osteosarcoma components and a significant increase in the Ki-67 index from 8% to 70%. These findings offer objective evidence of the tumor’s malignant progression. The clinical implications of this case present three key insights: (1) patients with recurrent PTB should undergo repeated biopsies to assess potential pathological grade progression; (2) in cases of clear malignant transformation, a comprehensive treatment strategy akin to those used for soft tissue sarcomas, such as the modified radical surgery combined with ifosfamide/cisplatin regimen employed in this case, should be considered; and (3) a long-term follow-up mechanism should be established to monitor the risk of late metastasis. Current research on PTB faces three major challenges: unclear molecular mechanisms, a lack of effective predictive markers, and inconsistent treatment protocols. Future studies should focus on multi-omics analysis, such as the MDM2/P53 pathway abnormalities identified in this case, and support the development of global case registries to facilitate personalized and precise treatment strategies.

## Data Availability

The original contributions presented in the study are included in the article/supplementary material. Further inquiries can be directed to the corresponding author.
